# Genome‐Wide DNA Methylation Changes Induced by High‐Fat Diet and Methyl Donor Supplementation in Female Lupus Models: An Exploratory Study

**DOI:** 10.1002/mnfr.70512

**Published:** 2026-06-11

**Authors:** Amanda Alves Ribeiro, Lucas Moura Carvalho, Jhulia Caroline Nunes Leal da Mota, Leticia Lobato Souza, Marcela Augusta de Souza Pinhel, Carla Barbosa Nonino, Bidossessi Wilfried Hounkpe, Walcy Paganelli Rosolia Teodoro, Nicolas Costa‐Fraga, Andrea Gonzales Izquierdo, Angel Diaz‐Lagares, Ana Belen Crujeiras, Carolina Ferreira Nicoletti

**Affiliations:** ^1^ Applied Physiology and Nutrition Research Group – School of Physical Education and Sport and Faculdade de Medicina FMUSP Universidade De Sao Paulo Brazil; ^2^ Center of Lifestyle Medicine, Faculdade de Medicina FMUSP Universidade De Sao Paulo Brazil; ^3^ Department of Internal Medicine, Ribeirão Preto Medical School University of São Paulo Brazil; ^4^ Division of Nutrition and Metabolism, Department of Health Sciences, Ribeirão Preto Medical School University of São Paulo Brazil; ^5^ Rheumatology Division, Hospital das Clinicas HCFMUSP, Faculdade de Medicina Universidade De São Paulo Sao Paulo Brazil; ^6^ Epigenomics Unit, Cancer Epigenomics, Translational Medical Oncology Group (ONCOMET); Clinical University Hospital & Health Research Institute of Santiago de Compostela. CIBERONC University of Santiago de Compostela, Santiago de Compostela Spain; ^7^ Epigenomics in Endocrinology and Nutrition Group, Epigenomics Unit, Health Research Institute of Santiago de Compostela (IDIS) University Clinical Hospital of Santiago (CHUS, SERGAS), Santiago de Compostela Spain; ^8^ Centro De Investigación Biomédica En Red Fisiopatología De La Obesidad y Nutrición (CIBERobn) Instituto De Salud Carlos III (ISCIII) Madrid Spain

**Keywords:** DNA methylation, epigenetic, folic acid, lupus, obesity, vitamin B12

## Abstract

This exploratory study investigated the interactive effects of dietary fat content and methyl‐donor supplementation on genome‐wide DNA methylation in adipose tissue of female lupus‐prone NZBWF1/J mice. Thirty mice were randomly assigned to four groups for 12 weeks in a 2 × 2 factorial design: standard diet (SD, *n* = 7), high‐fat diet (HFD, *n* = 7), standard diet supplemented (SDS, *n* = 8), and high‐fat diet supplemented (HFDS, *n* = 8). The standard diet provided 4.2 kcal/g and the high‐fat diet 6.6 kcal/g. Supplemented diets contained 8 mg/kg folic acid and 50 µg/kg vitamin B12. After a 10‐h fast, subcutaneous adipose tissue was collected for genome‐wide DNA methylation analysis using the Infinium Mouse Methylation BeadChip, followed by differential methylation and pathway enrichment analyses. The high‐fat diet induced a distinct methylation profile with 90 differentially methylated CpGs (*p* < 0.05, Δ*β* ≥ 0.05). Enrichment analyses did not identify significantly overrepresented pathways. Diet × supplementation interaction analysis identified 31 CpGs with significant interaction effects (*p* < 0.01). Pathway enrichment analysis revealed overrepresentation of the IL‐17 signaling pathway. Only one CpG overlapped between HFD‐associated and interaction‐associated loci, indicating largely distinct epigenetic responses to diet and supplementation. A high‐fat diet induces widespread epigenetic remodeling in adipose tissue, while folic acid and vitamin B12 supplementation modulates inflammatory epigenetic pathways in a diet‐dependent manner.

## Graphical abstract

A high‐fat diet induces widespread DNA methylation changes in adipose tissue in lupus‐prone mice, while methyl‐donor supplementation (folic acid and vitamin B12) modulates inflammatory pathways, including IL‐17 signaling, in a diet‐dependent manner.

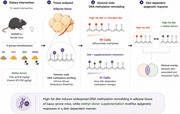

## 1 Introduction

Systemic lupus erythematosus (SLE) is a complex autoimmune disease that affects multiple organs and systems, presenting with diverse clinical manifestations [1]. While the precise etiology and pathogenic mechanisms of SLE remain incompletely understood, accumulating evidence highlights the critical roles of environmental factors, genetic predisposition, and epigenetic regulation triggering immune system dysregulation [2]. The immunopathogenesis of SLE is characterized by hyperactivation of B and T lymphocytes, chronic inflammation, immune complex deposition, and a breakdown of self‐tolerance [3].

Obesity has emerged as a significant modulator of immune responses and inflammation, playing a key role in the pathophysiology of several chronic diseases, including autoimmune disorders [4]. Notably, the prevalence of obesity among individuals with SLE is substantial, ranging from 30% to 40% [5, 6, 7]. Studies suggest that obesity may exacerbate autoimmune processes, contributing to worsened clinical manifestations, increased disease activity, and poorer prognoses [5, 8]. In fact, higher body mass index (BMI) has been positively associated with increased SLE disease activity, particularly among female patients, where excessive body weight correlates with more severe clinical symptoms compared to normal‐weight individuals [9, 10].

Epigenetic mechanisms, particularly DNA methylation, have been implicated in the pathogenesis of both SLE and obesity [11, 12]. DNA methylation, a well‐characterized epigenetic modification, involves the addition of a methyl group (CH3) to the 5′ position of cytosine residues in CpG dinucleotides, modulating gene expression by altering chromatin structure and accessibility [13, 14]. Aberrant DNA methylation patterns have been extensively reported in SLE, with disease activity and progression being inversely correlated with global DNA methylation levels [15]. Specifically, patients with active SLE display global DNA hypomethylation in peripheral lymphocytes, leading to overexpression of CD4+ T cells and subsequent immune dysregulation [16, 17, 18, 19].

Similarly, obesity is associated with epigenetic alterations in genes involved in metabolism, inflammation, and adipogenesis [20]. DNA hypomethylation of specific genes has been implicated in obesity pathogenesis, reinforcing the pro‐inflammatory state associated with excessive adiposity. Interestingly, emerging evidence suggests a potential epigenetic interplay between SLE and obesity, wherein obesity exacerbates SLE severity through shared mechanisms involving dysregulated DNA methylation. Collectively, evidence from experimental models indicates that SLE‐prone mice exposed to high‐fat diets may exhibit DNA methylation alterations, including hypomethylation patterns particularly described in immune cell populations, accompanied by enhanced immune activation and increased production of anti‐DNA autoantibodies [8, 21]. However, the extent to which these alterations represent global demethylation versus tissue‐specific epigenetic remodeling remains incompletely understood.

Moreover, DNA methylation is influenced by the availability of specific nutrients, particularly methyl donors such as folate and vitamin B12 [22]. These nutrients play essential roles in one‐carbon metabolism, providing methyl groups necessary for DNA methylation reactions [23, 24]. In vivo studies have demonstrated that diets with excessive or deficient methyl donors can significantly alter key metabolic intermediates, such as S‐adenosylmethionine (SAM) and S‐adenosylhomocysteine (SAH), leading to widespread epigenetic modifications [25, 26]. Notably, obesogenic diets supplemented with methyl donor nutrients have been shown to induce DNA hypermethylation, further modifying the epigenetic landscape [12].

Given the critical role of DNA methylation in both SLE and obesity, this exploratory study aims to investigate the DNA methylation profile in adipose tissue from SLE‐prone mice subjected to standard or high‐fat diets. Additionally, we will evaluate the potential impact of folic acid and vitamin B12 supplementation on genome‐wide DNA methylation profile.

## 2 Materials and Methods

### 2.1 Animals, Diets, and Experimental Design

A total of 30 female 8‐week‐old NZBWF1/J mice (initial body weight of 30 g) supplied by the Jackson Laboratory (Bar Harbor, ME) were housed in polysulfone cages (3–4 animals per cage) at controlled temperature (22 ± 2°C) and humidity (40%–60%) conditions with a 12:12 h light:darkness cycle. The mice received a standard diet (AIN‐93G) and water ad libitum for a 2‐week acclimation period.

On day 0 mice were randomly assigned (via Random.org) to one of four diets: 1. Standard Diet group (SD group, *n* = 7) that was fed a standard diet, 2. High‐fat diet group (HFD group, *n* = 7) that was fed a high‐fat diet, 3. standard diet supplemented group (SDS group, *n* = 8) that was fed a standard diet supplemented with folic acid and vitamin B12, and 4. high‐fat diet supplemented group (HFDS group, *n* = 8) that was fed a high‐fat diet supplemented with folic acid and vitamin B12.

All diets were purchased from Rhoster (Araçoiaba da Serra, SP, Brazil). The standard diet contained 4.2 kcal/g in dry weight, whereas HFD contained 6.6 kcal/g (Table S1). The supplemented diets differed from their base diets by including 8 mg/kg of folic acid and 50 µg/kg of vitamin B12. The supplementation doses were selected based on previous rodent studies investigating methyl donor modulation and DNA methylation dynamics, in which similar concentrations were shown to alter one‐carbon metabolism without inducing toxicity. These doses are considered nutritionally enhanced rather than pharmacological, aiming to modulate physiological methyl donor availability [27].

The diets were stored at 4°C until they were provided to the animals. The food was replenished three times per week. Before that, the remaining food in each cage was weighed. After cleaning, freshly weighed food was added to the cages. Food intake was determined by calculating the difference between the total food provided and the residual amount in each cage, normalized to the number of animals per cage. Intake assessments were conducted separately for each experimental group.

Body weight was monitored three times per week, and the mean value for each animal was calculated for subsequent analyses. Weighing was performed on the same days each week to ensure uniformity in the data collection process. After 12 weeks of ad libitum dietary treatment, the animals were euthanized using ketamine/xylazine overdoses following a 10‐h fast. Samples of adipose depots (subcutaneous) were carefully dissected and stored immediately at −80°C. All the procedures performed agreed with guidelines for the care and use of experimental animals and study protocol was approved by Institutional Animal Experimentation Ethics Committee (No. 1695/2021) of the School of Medicine—University of Sao Paulo.

### 2.2 Statistical Analysis of Phenotypic Data

Numerical variables were described using mean ± standard deviation. The Shapiro–Wilk test verified the normality of data distribution. Generalized Estimating Equations statistical significance was set at 5% (*p* < 0.05). Analyses were performed using SPSS version 22.0 (Inc., Chicago, IL).

### 2.3 DNA Methylation Analysis

DNA was extracted using the DNeasy Blood and Tissue Kit (Qiagen), followed by bisulfite treatment using the EZ DNA Methylation‐Gold kit (Zymo Research), according to the manufacturer's instructions. Bisulfite‐converted DNA was then amplified and hybridized to the Infinium Mouse Methylation BeadChip (Illumina, CA, USA), and scanned using the Illumina iScan system (Illumina, CA, USA), following the manufacturer's protocols.

### 2.4 Quality Control and Preprocessing

Raw IDAT intensity files were imported into R (v4.5) and processed using the *minfi* package (v1.54.1) [28]. Arrays were normalized via quantile normalization to correct for systematic differences between Type I and Type II probes. Sample integrity was evaluated using internal control probes and by assessing concordance between reported and inferred sex. Probes were excluded if they presented a detection *p*‐value > 0.01 in any sample or contained single nucleotide polymorphisms at the single‐base extension or CpG interrogation site. Sample‐level quality control included inspection of control probes, hierarchical clustering, and principal component analysis (PCA) to identify potential outliers and assess potential batch effects. No systematic batch structure was identified. DNA methylation levels were quantified as *β*‐values ranging from 0 (fully unmethylated) to 1 (fully methylated).

### 2.5 Probe‐Wise Differential Methylation Analysis

Single‐CpG differential methylation was assessed using the limma package (v3.64.3). The design matrix included diet, supplementation, and their interaction term (diet × supplementation), allowing estimation of both main and interaction effects on DNA methylation levels. Linear models were fitted to M‐values to improve statistical performance, and empirical Bayes moderation (eBayes) was applied to stabilize variance estimates. Differentially methylated CpGs were defined as loci with *p *< 0.05 and an absolute methylation difference (Δ*β*) ≥ 0.05 between comparison groups. Body weight and caloric intake were not included as covariates because these variables represent biological outcomes of the dietary intervention rather than independent confounding factors. For the interaction analysis, CpGs with *p *< 0.01 were considered nominally significant.

### 2.6 Gene‐Set Enrichment Analysis

Functional annotation of genes associated with differentially methylated CpGs was performed by mapping CpG sites to gene symbols using the Illumina Mouse Methylation array annotation. Gene‐set enrichment analysis was conducted using both the KEGG and Gene Ontology (GO) databases through the *clusterProfiler* R package to explore biological pathways associated with diet × supplementation interaction CpGs.

## 3 Results

**FIGURE 1:**
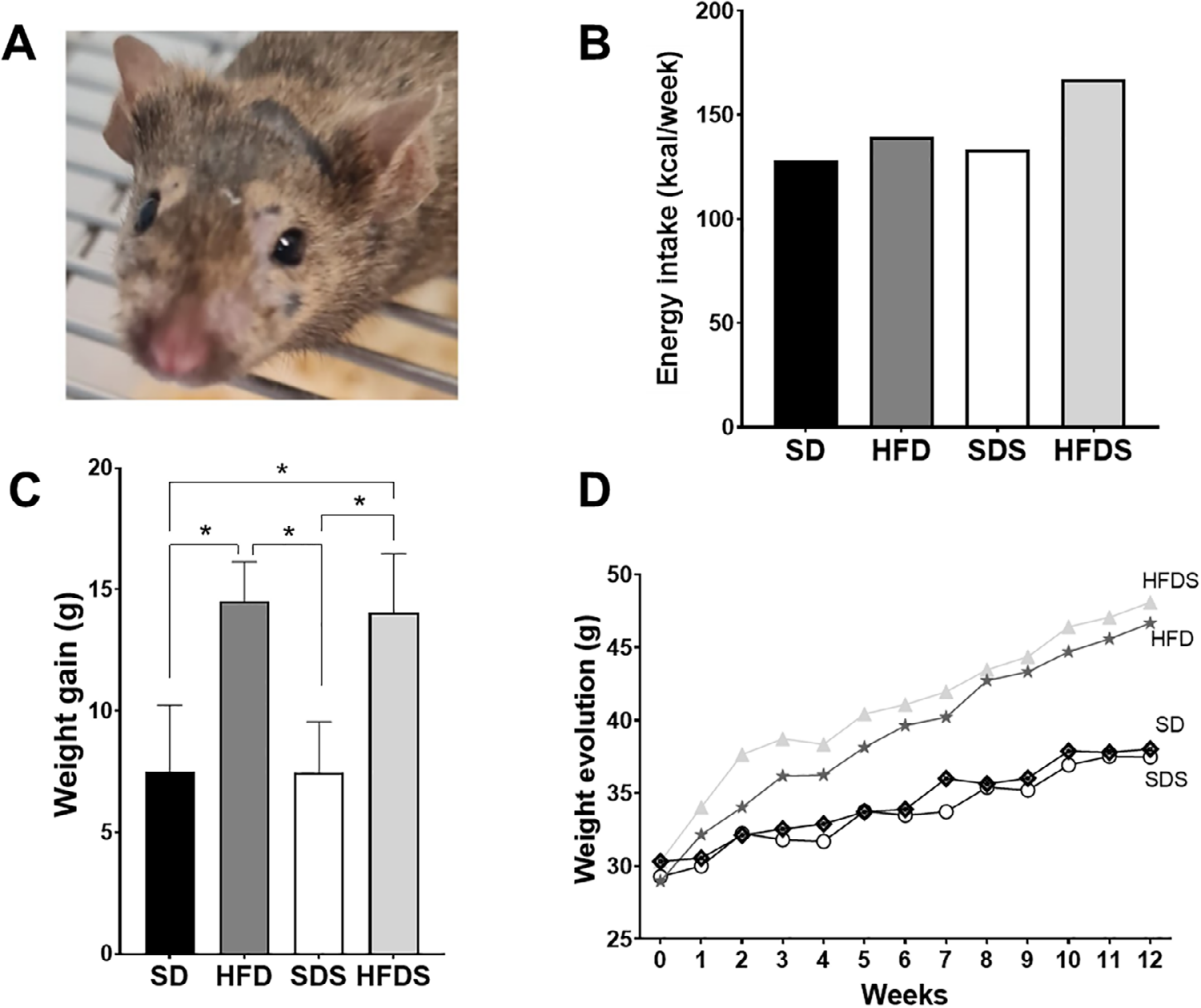
Effects of dietary interventions on clinical and metabolic parameters in NZBWF1/J mice. (A) Representative image showing malar rash, a clinical manifestation consistent with SLE development. (B) Total energy intake (kcal/week) during the 12‐week intervention. Consumption was measured per cage, and therefore no statistical analysis was performed. (C) Weight gain (g) after 12 weeks of dietary intervention. (D) Body weight evolution (g) over the 12‐week experimental period. Data are presented as mean ± SEM. *p* < 0.05. SD: standard diet; HFD: high‐fat diet; SDS: standard diet + supplement; HFDS: high‐fat diet + supplement. Sample sizes: SD (*n* = 7), HFD (*n* = 7), SDS (*n* = 8), and HFDS (*n* = 8). All replicates correspond to independent biological samples (individual animals). Longitudinal body weight analysis was performed using Generalized Estimating Equations (GEE).

All animals completed the experimental period, and clinical signs of SLE development were observed across all groups. A notable manifestation was the presence of *rash malar* (Figure 1A). This finding reinforces the relevance of the NZBWF1/J model in studying SLE pathophysiology, as it closely mimics human disease features. Total caloric intake over the course of the 12 weeks according to groups is demonstrated in Figure 1B. Considering that the consumption was assessed per cage rather than per animal, no statistical analysis was performed. As expected, HFD induced an obese phenotype in both HFD and HFDS groups (Figure 1C). After 12 weeks, weight gain in HFD (14.5±1.6 g) and HFDS (14±2.4 g) groups was higher than in the SD (7.5±2.7 g) and SDS (7.4±2.1 g) groups (all *p *< 0.05). No relevant differences were found between the weight gain among mice fed the standard or supplemented diets (Figure 1D).

### 3.1 Global Effects of High‐Fat Diet

**FIGURE 2:**
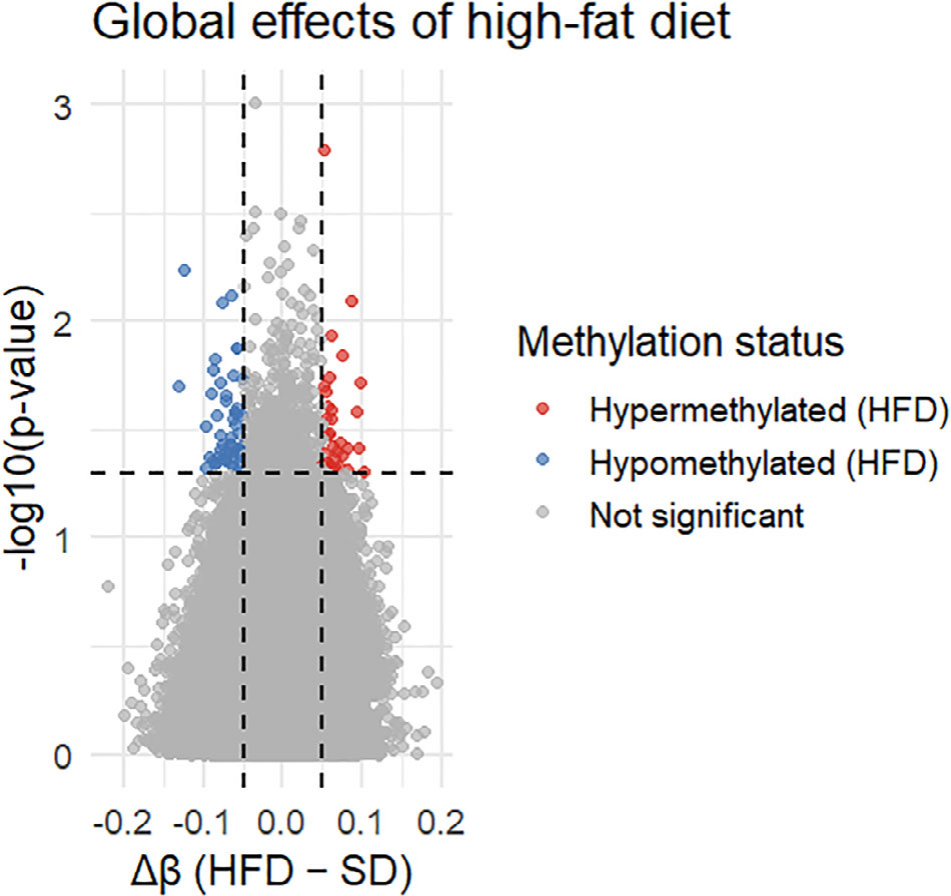
Global effects of high‐fat diet on genome‐wide DNA methylation. Volcano plot representing differentially methylated CpG sites (DMCpGs) between the high‐fat diet (HFD) and standard diet (SD) groups. The *x*‐axis shows the methylation difference (Δ*β*, HFD − SD), and the *y*‐axis displays −log10(*p*‐value). CpGs classified as hypermethylated in the HFD group are shown in red, while hypomethylated CpGs are shown in blue. Grey dots represent CpGs that did not reach statistical significance. Dashed vertical and horizontal lines indicate the thresholds used for differential methylation. Sample sizes: SD (*n* = 7), HFD (*n* = 7), SDS (*n* = 8), and HFDS (*n* = 8). All samples represent independent biological replicates.

Genome‐wide DNA methylation analysis revealed a diet‐specific epigenetic signature driven by HFD exposure. Comparative analysis between HFD and SD groups identified 90 differentially methylated CpGs (DMCpGs) (*p*< 0.05, Δβ ≥ 0.05). Among these loci, 36 CpGs were hypermethylated and 54 were hypomethylated in the HFD group, indicating that both directions of methylation change contribute to the diet‐induced epigenetic response (Figure 2). Table 1 shows the top 15 DMCpGs sites (considering Δ*β* values). Functional enrichment analysis using both KEGG and Gene Ontology databases did not identify pathways reaching statistical significance after multiple testing correction. The associated genes include proteins involved in transcriptional regulation, cellular signaling, and structural or membrane‐associated processes, as well as several predicted or poorly characterized loci. This pattern suggests that HFD‐associated methylation alterations are functionally heterogeneous and broadly distributed across multiple genomic contexts rather than concentrated within specific canonical biological pathways.

**TABLE 1:** Top 15 differentially methylated CpG sites identified between high‐fat diet (HFD) and standard diet (SD) groups.

CpG ID	Gene	Δβ (HFD − SD)	Direction (HFD vs. SD)	*p*‐value
cg28495593_BC11	—	−0.09	Hypomethylated in HFD	0.0439
cg31019775_TC21	—	−0.09	Hypomethylated in HFD	0.0167
cg31529292_TC21	Atxn1	−0.10	Hypomethylated in HFD	0.0471
cg34415276_BC11	—	−0.09	Hypomethylated in HFD	0.0422
cg36259492_TC21	Kank1	0.10	Hypermethylated in HFD	0.0192
cg38974905_BC21	—	−0.12	Hypomethylated in HFD	0.0058
cg41573042_BC11	Alpl	0.09	Hypermethylated in HFD	0.0263
cg42155381_BC21	—	−0.09	Hypomethylated in HFD	0.0450
cg42984005_TC21	Rbakdn	−0.13	Hypomethylated in HFD	0.0200
cg43264573_TC21	Mest	0.09	Hypermethylated in HFD	0.0080
cg43652664_BC21	A430078I02Rik	0.10	Hypermethylated in HFD	0.0385
cg43967350_BC21	Lpar5	−0.10	Hypomethylated in HFD	0.0302
cg44903935_BC21	—	0.10	Hypermethylated in HFD	0.0490
cg47578653_TC21	Gria3	−0.09	Hypomethylated in HFD	0.0460
cg48221478_TC11	Gm3376	−0.09	Hypomethylated in HFD	0.0216

*Note: Δβ* represents the difference in methylation levels between groups (HFD − SD). Positive values indicate higher methylation levels in HFD, whereas negative values indicate lower methylation levels in HFD.

### 3.2 Diet × Supplementation Interaction

**FIGURE 3:**
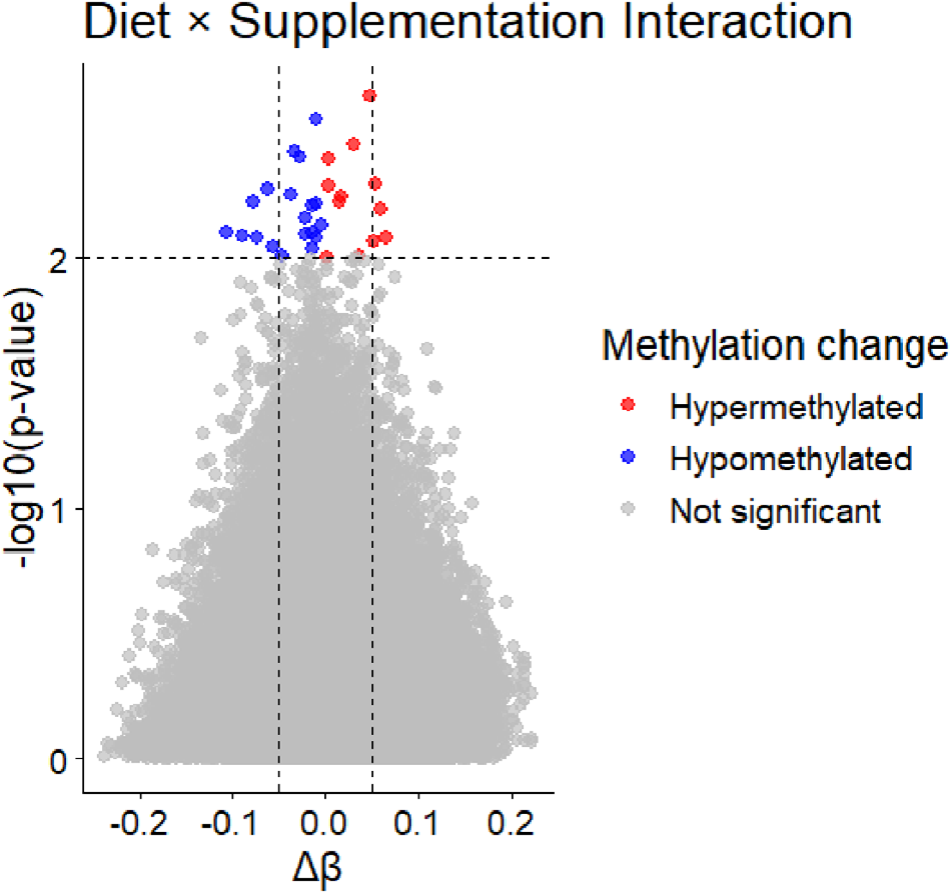
Diet × supplementation interaction effects on genome‐wide DNA methylation. Volcano plot illustrates CpG sites exhibiting significant diet × supplementation interaction effects on DNA methylation. The *x*‐axis represents the log2 fold change, and the *y*‐axis shows −log10(*p*‐value). CpGs classified as hypermethylated are shown in red and hypomethylated CpGs in blue, while grey dots represent CpGs that were not statistically significant. Dashed vertical and horizontal lines indicate the thresholds used to identify differentially methylated CpGs (*p* < 0.01 and |log2FC| > 1). Sample sizes: SD (*n* = 7), HFD (*n* = 7), SDS (*n* = 8), and HFDS (*n* = 8). All samples correspond to independent biological replicates.

A total of 31 CpGs exhibited nominally significant diet × supplementation interaction effects (*p* < 0.01). Among these loci, 12 CpGs showed higher methylation levels in the HFDS group relative to SD, whereas 19 CpGs showed lower methylation levels, indicating that the epigenetic response to supplementation is strongly dependent on dietary context. The magnitude of methylation changes across these loci was generally modest but biologically relevant, with Δ*β* values ranging from approximately –0.03 to +0.05, consistent with typical effect sizes observed in epigenome‐wide studies. Volcano plot analysis highlighted CpGs showing nominally significant interaction effects (*p* < 0.01), indicating loci where supplementation modifies methylation patterns depending on dietary context (Figure 3). To highlight the most biologically relevant loci, the 15 CpGs with the largest absolute Δ*β* values were selected (Table 2). Notably, several CpGs were located within promoter‐associated regions or CpG islands, suggesting potential regulatory implications for gene expression. KEGG pathway enrichment analysis identified significant overrepresentation of the IL‐17 signaling pathway (adjusted *p* = 0.032) among genes associated with CpGs exhibiting diet × supplementation interaction effects. This pathway is a key mediator of inflammatory responses and has been widely implicated in autoimmune diseases, including systemic lupus erythematosus. The enrichment suggests that micronutrient supplementation may influence diet‐dependent epigenetic regulation of inflammatory signaling pathways in adipose tissue. Collectively, these findings indicate that vitamin supplementation exerts context‐dependent epigenetic modulation, with its methylation effects varying according to dietary exposure. This interaction pattern suggests that nutritional supplementation may partially reshape diet‐induced epigenetic signatures in adipose tissue.

**TABLE 2:** Top 15 CpG sites showing nominally significant diet × supplementation interaction effects.

CpG ID	Gene	Δ*β*	*p*‐value
cg30058259_BC11	Cd300lg	0.05	0.0051
cg33469136_BC11	Peg13 / Trappc9	−0.11	0.0079
cg33694350_TC21	Slc2a13	−0.04	0.0056
cg35550539_TC11	—	0.05	0.0085
cg37506265_BC21	Gm4793 / Nav1	−0.09	0.0082
cg38781187_BC11	Mpped2	−0.07	0.0084
cg40211529_BC11	—	−0.05	0.0098
cg40922250_BC21	Ctnnal1	−0.08	0.0059
cg41121889_BC11	Gm12610	0.04	0.0097
cg42984005_TC21	Rbakdn	−0.03	0.0038
cg43999659_TC21	Tspan9	0.07	0.0083
cg45816167_BC11	Gm3336 / Gm34255	0.06	0.0064
cg38781187_BC11	Mpped2	−0.07	0.0084
cg40211529_BC11	—	−0.05	0.0098
cg40922250_BC21	Ctnnal1	−0.08	0.0059

*Note: Δβ* represents the difference in methylation levels between groups (HFD − SD). Positive values indicate higher methylation levels in HFD, whereas negative values indicate lower methylation levels in HFD.

### 3.3 Overlap of Differentially Methylated CpGs

**FIGURE 4:**
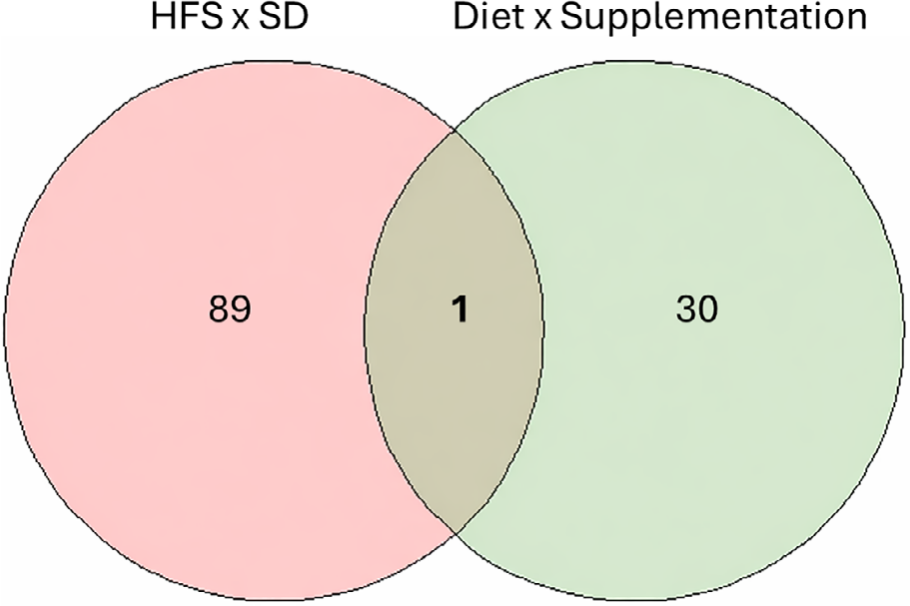
Overlap of differentially methylated CpGs between dietary and interaction effects. Venn diagram showing the overlap between CpG sites differentially methylated in response to high‐fat diet (HFD vs. SD) and those associated with the diet × supplementation interaction effect. A total of 89 CpGs were unique to the HFD comparison, 30 CpGs were specific to the interaction effect, and 1 CpG was shared between both conditions. Sample sizes: SD (*n* = 7), HFD (*n* = 7), SDS (*n* = 8), and HFDS (*n* = 8). Differentially methylated CpGs were defined according to the statistical criteria described in the Methods section.

Comparative analysis between diet‐associated CpGs (HFD vs. SD) and diet × supplementation interaction CpGs revealed minimal overlap between the two sets (Figure 4). Only one CpG site was shared between the HFD‐associated methylation signature and the diet × supplementation interaction loci. In contrast, the vast majority of CpGs were condition‐specific, with 89 sites unique to the HFD comparison and 30 sites unique to the interaction analysis. This unique CpG site (cg42984005_TC21) was annotated to the Rbakdn gene and located within the gene body region near the transcription start site (tss_body) in an OpenSea CpG context. This limited overlap suggests that diet‐induced methylation changes and supplementation‐modulated methylation responses largely occur at distinct genomic loci, indicating that micronutrient supplementation may reshape epigenetic patterns through mechanisms that are not simply the reversal of diet‐induced alterations.

## 4 Discussion

In the present study, all animals developed hallmark features of SLE, including malar rash, confirming the robustness and translational relevance of the NZBWF1/J model. As expected, the HFD promoted marked weight gain, consistent with its obesogenic effect. In contrast, micronutrient supplementation did not affect body weight, suggesting that its potential effects may occur primarily at the epigenetic level rather than through observable phenotypic changes.

Previous studies have shown that obesity is linked to a chronic low‐grade inflammatory state, driven by increased levels of proinflammatory cytokines such as TNF‐α and IL‐6 [29, 30]. This inflammatory condition may worsen autoimmunity, accelerating both the severity and progression of SLE [31, 32]. Our genome‐wide DNA methylation analysis supports this idea, showing that HFD induced a distinct epigenetic profile. Almost one hundred CpGs were differentially methylated, with a balanced distribution of hyper‐ and hypomethylated sites, indicating broad reprogramming of the epigenome. However, enrichment analyses did not identify specific canonical pathways associated with these CpGs, suggesting that HFD‐induced methylation alterations are widely distributed across the genome rather than concentrated in specific biological pathways. These findings are consistent with the concept that obesogenic environments may promote heterogeneous epigenetic remodeling, which affects multiple biological processes simultaneously.

The HFD used in this study contained higher amounts of hydrogenated vegetable fat, which may have contributed to epigenetic remodeling and the inflammatory phenotypes observed. These findings are consistent with previous murine studies showing that high‐fat diets alter DNA methylation and gene expression in adipose tissue and liver, affecting insulin signaling and inflammatory pathways [33, 34, 35].

In humans, controlled interventions have demonstrated that dietary lipid composition directly impacts the epigenome of metabolically active tissues. For instance, in the LIPOGAIN study, overfeeding either saturated or polyunsaturated fatty acids induced distinct DNA methylation signatures in subcutaneous adipose tissue, modulating genes involved in insulin signaling and inflammatory pathways [36]. Epigenetic alterations in response to high‐fat diets have also been reported in skeletal muscle, where short‐term overfeeding induced widespread methylation changes in genes linked to energy metabolism and insulin sensitivity [37]. Similarly, Gillberg et al. [38] showed that only five days of high‐fat overfeeding were sufficient to alter the expression of genes such as *INSR* and *IRS2* in adipose tissue, reinforcing the role of dietary fats in driving epigenetic remodeling associated with insulin resistance. Collectively, these findings demonstrate that the mechanisms described in murine models are also evident in humans, supporting the view that high‐fat diets promote pro‐inflammatory phenotypes and metabolic dysregulation through epigenetic pathways.

Interestingly, micronutrient supplementation showed strong effects on DNA methylation, but only in the context of animals being fed with HFD. Thirty‐one CpGs exhibited significant diet × supplementation interaction effects, indicating that the epigenetic response to methyl‐donor supplementation depends strongly on dietary context. This observation suggests that supplementation does not exert uniform epigenetic effects but rather interacts with the metabolic environment created by the diet. Such context‐dependent epigenetic modulation may reflect alterations in one‐carbon metabolism and methyl group availability under obesogenic conditions. KEGG pathway enrichment analysis revealed significant overrepresentation of the IL‐17 signaling pathway among CpGs affected by the diet × supplementation interaction. This finding is particularly relevant in the context of SLE pathogenesis. IL‐17 is a pro‐inflammatory cytokine mainly produced by Th17 cells and has been widely implicated in autoimmune diseases, including systemic lupus erythematosus. Increased IL‐17 activity has been associated with enhanced autoantibody production, tissue inflammation, and disease severity in lupus patients and experimental models. Our findings therefore suggest that methyl‐donor supplementation may influence the epigenetic regulation of inflammatory pathways linked to Th17 activity, particularly under obesogenic conditions.

Numerous studies have demonstrated that micronutrient supplementation can influence phenotypic variables such as adiposity, glucose metabolism, and lipid metabolism in response to HFD intake [39, 40, 41]. In parallel, evidence indicates that nutritional interventions exert beneficial effects on DNA methylation, both at global and gene‐specific levels, particularly in metabolic pathways [42]. Dietary nutrients and bioactive compounds may modulate DNA methylation patterns by affecting the activity of enzymes involved in methylation reactions or by altering the availability of methyl donors, thereby influencing metabolic regulation [42]. In this context, a recent systematic review and meta‐analysis showed that folic acid supplementation can modify global DNA methylation levels, with higher doses producing effects distinct from those observed with lower doses [43].

Together, these findings suggest that diet‐induced epigenetic modifications may contribute to metabolic adaptations and potentially to clinical heterogeneity in complex diseases such as SLE. However, despite consistent evidence in metabolic models, important gaps remain regarding the impact of these nutritional and epigenetic interactions in autoimmune disease models.

When comparing the CpGs altered by HFD with those affected by the diet × supplementation interaction, only one CpG site overlapped between the two analyses. This CpG (cg42984005_TC21) was annotated to the Rbakdn gene and located in the gene body region near the transcription start site in an OpenSea CpG context. The minimal overlap between these datasets suggests that diet‐induced methylation changes and supplementation‐responsive CpGs largely occur at distinct genomic loci. This observation indicates that micronutrient supplementation may reshape epigenetic patterns through mechanisms that are distinct from, rather than simply reversing, diet‐induced methylation changes.

Although SLE is primarily an immune‐mediated disease, our epigenetic analyses were restricted to subcutaneous adipose tissue. Adipose tissue is increasingly recognized as an immunometabolic organ capable of modulating systemic inflammation through adipokine secretion and immune cell infiltration. Moreover, adipose tissue represents a heterogeneous compartment composed of adipocytes, stromal cells, and infiltrating immune cells, which may influence the observed methylation profiles. However, methylation patterns identified in adipose tissue may not fully reflect epigenetic alterations occurring in classical immune cell subsets such as T and B lymphocytes, which are central to SLE pathogenesis. Future studies integrating cell‐specific epigenetic analyses into immune compartments will be necessary to clarify the systemic relevance of the observed methylation changes.

This study has several limitations. First, the sample size was relatively modest (*n* = 7–8 per group), which may limit statistical power in genome‐wide methylation analyses and increase the possibility of type II error, particularly when evaluating diet × supplementation interaction effects. Differentially methylated CpGs were identified using nominal *p*‐value thresholds, and most loci did not remain significant after multiple testing correction. Therefore, the methylation findings should be interpreted as exploratory and hypothesis‐generating rather than definitive. Second, our analyses were restricted to DNA methylation and did not include parallel transcriptomic or proteomic profiling, limiting direct inference regarding functional consequences at the gene expression or protein level. Third, although the NZBWF1/J strain is a well‐characterized spontaneous lupus model and clinical manifestations such as malar rash were observed in all animals, circulating markers of disease activity (e.g., anti‐dsDNA antibodies or renal histopathology) were not quantified. Consequently, we cannot directly associate methylation patterns with quantitative measures of lupus severity. Additionally, food intake was measured at the cage level rather than individually, precluding precise assessment of individual caloric consumption. Despite these limitations, a major strength of the study lies in the genome‐wide methylation approach, which provides a comprehensive framework for hypothesis generation. These findings support the design of future integrative studies combining epigenomic, transcriptomic, and immunophenotypic analyses to better understand diet‐ and supplementation‐induced epigenetic modulation in autoimmune disease contexts [35, 44].

## 5 Conclusion

A high‐fat diet induces widespread DNA methylation changes across the adipose tissue epigenome, whereas folic acid and vitamin B12 supplementation modulates these effects in a diet‐dependent manner. This pattern demonstrates that nutritional interventions can act as adaptive modulators of the epigenome, even without affecting macroscopically observable phenotypes, such as body weight. Supplementation influenced CpGs associated with inflammatory signaling pathways, including IL‐17 signaling, suggesting that methyl‐donor availability may shape epigenetic regulation of immune pathways under obesogenic conditions. These findings underscore the potential of personalized nutritional strategies to restore metabolic and immune homeostasis and provide a foundation for future integrative studies linking the methylome, transcriptome, and immune phenotypes in models of autoimmune disease.

## Funding

This study was supported by São Paulo Research Foundation (FAPESP), grants number #2020/01893‐2, #2020/15126‐3, #2021/09753‐8.

## Conflicts of Interest

The authors declared no potential conflicts of interest.

## Supporting information




**Supporting File**: mnfr70512‐sup‐0001‐TableS1.docx.

## Data Availability

The datasets used and/or analyzed during the current study are available from the corresponding author on reasonable request.

## References

[1] P. C. Bagare , A. Borle , P. Baluni , G. G. Ekbote , and S. Sangale , “Clinical Profile and Outcomes of Patients With Systemic Lupus Erythematosus,” Cureus 16, no. 9 (2024):e68541, 10.7759/cureus.68541. 39364459 PMC11448960

[2] M. A. Ameer , H. Chaudhry , J. Mushtaq , et al., “An Overview of Systemic Lupus Erythematosus (SLE) Pathogenesis, Classification, and Management,” Cureus 14 (2022): 30330, 10.7759/CUREUS.30330. PMC9662848 36407159

[3] D. B. Catharine Cunha , R. Mira , L. Guanais Soriano , C. Bittencourt Cunha , and R. Campos Nascimento , “Lupus Eritematoso Sistêmico: Novos Paradigmas e Manejo no Atendimento Emergencial – Revisão de Literatura,” Revista Científica Hospital Santa Izabel 4 (2020): 57–62.

[4] T. V. Rohm , D. T. Meier , J. M. Olefsky , and M. Y. Donath , “Inflammation in Obesity, Diabetes, and Related Disorders,” Immunity 55 (2022): 31–55, 10.1016/j.immuni.2021.12.013. 35021057 PMC8773457

[5] M. Kono , Y. Nagafuchi , H. Shoda , and K. Fujio , “The Impact of Obesity and a High‐Fat Diet on Clinical and Immunological Features in Systemic Lupus Erythematosus,” Nutrients 13 (2021): 504, 10.3390/nu13020504. 33557015 PMC7913625

[6] P. Teh , B. Zakhary , and V. K. Sandhu , “The Impact of Obesity on SLE Disease Activity: Findings From the Southern California Lupus Registry (SCOLR),” Clinical Rheumatology 38 (2019): 597–600, 10.1007/s10067-018-4336-3. 30357495

[7] E. B. Taylor , “The Complex Role of Adipokines in Obesity, Inflammation, and Autoimmunity,” Clinical Science 135 (2021): 731–752, 10.1042/CS20200895. 33729498 PMC7969664

[8] N. Hanna Kazazian , Y. Wang , A. Roussel‐Queval , et al., “Lupus Autoimmunity and Metabolic Parameters Are Exacerbated Upon High Fat Diet‐Induced Obesity due to TLR7 Signaling,” Frontiers in Immunology 10 (2019): 2015–2015, 10.3389/fimmu.2019.02015. 31552019 PMC6738575

[9] L. M. Carvalho , B. G. Carvalho , L. L. Souza , J. C. da Mota , A. A. Ribeiro , and C. F. Nicoletti , “Obesity as an Aggravating Factor of Systemic Lupus Erythematosus Disease: What We Already Know and What We Must Explore. A Rapid Scoping Review,” Nutrition 128 (2024): 112559, 10.1016/j.nut.2024.112559. 39244807

[10] S. Chaiamnuay , A. M. Bertoli , M. Fernández , et al., “The Impact of Increased Body Mass Index on Systemic Lupus Erythematosus,” Journal of Clinical Rheumatology 13 (2007): 128–133, 10.1097/RHU.0b013e3180645865. 17551377

[11] B. Richardson , “Epigenetically Altered T Cells Contribute to Lupus Flares,” Cells 8 (2019): 127, 10.3390/CELLS8020127. 30764520 PMC6406295

[12] A. la Cava , “Molecular Characterization and Expression of *SPP1*, *LAP3* and *LCORL* and Their Association With Growth Traits in Sheep,” Genes (Basel) 10 (2019): 405, 10.3390/GENES10050405. 31416156 PMC6723280

[13] S. Hiramatsu , K. S. Watanabe , S. Zeggar , et al., “Regulation of Cathepsin E Gene Expression by the Transcription Factor Kaiso in MRL/Lpr Mice Derived CD4+ T Cells,” Scientific Reports 9 (2019): 3054, 10.1038/S41598-019-38809-Y. 30816218 PMC6395770

[14] S. S. S. Boyanapalli and A. N. T. Kong , ““Curcumin, the King of Spices”: Epigenetic Regulatory Mechanisms in the Prevention of Cancer, Neurological, and Inflammatory Diseases,” Current Pharmacology Reports 1 (2015): 129–139, 10.1007/s40495-015-0018-x. 26457241 PMC4596544

[15] Y. Zhan , Y. Guo , and Q. Lu , “Aberrant Epigenetic Regulation in the Pathogenesis of Systemic Lupus Erythematosus and Its Implication in Precision Medicine,” Cytogenetic and Genome Research 149 (2016): 141–155, 10.1159/000448793. 27607472

[16] C. M. Hedrich , “Epigenetics in SLE,” Current Rheumatology Reports 19 (2017): 58, 10.1007/s11926-017-0685-1. 28752494 PMC5532407

[17] A. E. A. Surace and C. M. Hedrich , “The Role of Epigenetics in Autoimmune/Inflammatory Disease,” Frontiers in immunology 10 (2019): 1525, 10.3389/fimmu.2019.01525. 31333659 PMC6620790

[18] P. L. Meroni and A. E. Penatti , “Epigenetics and Systemic Lupus Erythematosus: Unmet Needs,” Clinical Reviews in Allergy & Immunology 50 (2016): 367–376, 10.1007/s12016-015-8497-4. 26206675

[19] S. Pyfrom , B. Paneru , J. J. Knox , et al., “The Dynamic Epigenetic Regulation of the Inactive X Chromosome in Healthy human B Cells is Dysregulated in Lupus Patients,” PNAS 118 (2021): 2024624118, 10.1073/PNAS.2024624118/-/DCSUPPLEMENTAL. PMC8214693 34103397

[20] L. Chiricosta , S. Silvestro , A. Gugliandolo , et al., “Extracellular Vesicles of Human Periodontal Ligament Stem Cells Contain MicroRNAs Associated to Proto‐Oncogenes: Implications in Cytokinesis,” Frontiers in Genetics 11 (2020): 1–9, 10.3389/fgene.2020.00582. 32582296 PMC7287171

[21] Y. Liu , Y. Yu , G. Matarese , and A. L. Cava , “Cutting Edge: Fasting‐Induced Hypoleptinemia Expands Functional Regulatory T Cells in Systemic Lupus Erythematosus,” Journal of Immunology 188 (2012): 2070–2073, 10.4049/jimmunol.1102835. PMC3288569 22291185

[22] A. Mahajan , D. Sapehia , R. Bagga , and J. Kaur , “Different Dietary Combinations of Folic Acid and Vitamin B12 in Parental Diet Results in Epigenetic Reprogramming of IGF2R and KCNQ1OT1 in Placenta and Fetal Tissues in Mice,” Molecular Reproduction and Development 88 (2021): 437–458, 10.1002/mrd.23477. 34008284

[23] S. Barua , S. Kuizon , W. T. Brown , and M. A. Junaid , “DNA Methylation Profiling at Single‐Base Resolution Reveals Gestational Folic Acid Supplementation Influences the Epigenome of Mouse Offspring Cerebellum,” Frontiers in Neuroscience 10 (2016): 168, 10.3389/fnins.2016.00168. 27199632 PMC4854024

[24] A. Chango and I. P. Pogribny , “Considering Maternal Dietary Modulators for Epigenetic Regulation and Programming of the Fetal Epigenome,” Nutrients 7 (2015): 2748–2770, 10.3390/nu7042748. 25875118 PMC4425171

[25] S. Vordenbäumen , A. Sokolowski , A. Rosenbaum , et al., “Methyl Donor Micronutrients, CD40‐ligand Methylation and Disease Activity in Systemic Lupus Erythematosus: A Cross‐Sectional Association Study,” Lupus 30 (2021): 1773–1780, 10.1177/09612033211034559. 34284675 PMC8564257

[26] K. Pesqueda‐Cendejas , M. Rivera‐Escoto , M. R. Meza‐Meza , et al., “Nutritional Approaches to Modulate Cardiovascular Disease Risk in Systemic Lupus Erythematosus: A Literature Review,” Nutrients 15 (2023): 1036, 10.3390/NU15041036. 36839394 PMC9958972

[27] J. C. N. L. da Mota , A. A. Ribeiro , L. M. Carvalho , et al., “Impact of Methyl‐Donor Micronutrient Supplementation on DNA Methylation Patterns: A Systematic Review and Meta‐Analysis of in Vitro, Animal, and Human Studies,” Lifestyle Genomics 16, no. 1 (2023): 192–213, 10.1159/000533193. 37935134

[28] M. J. Aryee , A. E. Jaffe , H. Corrada‐Bravo , et al., “Minfi: A Flexible and Comprehensive Bioconductor Package for the Analysis of Infinium DNA Methylation Microarrays,” Bioinformatics 30 (2014): 1363–1369, 10.1093/bioinformatics/btu049. 24478339 PMC4016708

[29] “Changes of Serum IL‐6, IL‐10 and TNF‐α Levels in Patients With Systemic Lupus Erythematosus and Their Clinical Value—PubMed,” available at https://pubmed.ncbi.nlm.nih.gov/34017450/. PMC8129414 34017450

[30] U. De la Cruz‐Mosso , T. García‐Iglesias , R. Bucala , et al., “MIF Promotes a Differential Th1/Th2/Th17 Inflammatory Response in Human Primary Cell Cultures: Predominance of Th17 Cytokine Profile in PBMC From Healthy Subjects and Increase of IL‐6 and TNF‐α in PBMC From Active SLE Patients,” Cellular Immunology 324 (2018): 42–49. 29397904 10.1016/j.cellimm.2017.12.010

[31] G. Matarese , “The Link Between Obesity and Autoimmunity,” Science 379 (2023): 1298–1300, 10.1126/science.ade0113. 36996218

[32] E. B. Taylor , “The Complex Role of Adipokines in Obesity, Inflammation, and Autoimmunity,” Clinical Science 135 (2021): 731–752, https://portlandpress.com/clinsci/article/135/6/731/228093/The‐complex‐role‐of‐adipokines‐in‐obesity. 33729498 10.1042/CS20200895 PMC7969664

[33] J. de Jesus Simão , A. F. de Sousa Bispo , V. T. G. Plata , L. M. Armelin‐Correa , and M. I. C. Alonso‐Vale , “Fish Oil Supplementation Mitigates High‐Fat Diet‐Induced Obesity: Exploring Epigenetic Modulation and Genes Associated With Adipose Tissue Dysfunction in Mice,” Pharmaceuticals 17 (2024): 861. 39065712 10.3390/ph17070861 PMC11280081

[34] A. Abbas , T. Witte , W. L. Patterson , et al., “Epigenetic Reprogramming Mediated by Maternal Diet Rich in Omega‐3 Fatty Acids Protects From Breast Cancer Development in F1 Offspring,” Frontiers in Cell and Developmental Biology 9 (2021): 682593, 10.3389/fcell.2021.682593. 34179012 PMC8222782

[35] M. R. Keleher , R. Zaidi , L. Hicks , et al., “A High‐fat Diet Alters Genome‐wide DNA Methylation and Gene Expression in SM/J Mice,” BMC Genomics 19, no. 1 (2018): 888, 10.1186/s12864-018-5327-0. 30526554 PMC6286549

[36] A. Perfilyev , I. Dahlman , L. Gillberg , et al., “Impact of Polyunsaturated and Saturated Fat Overfeeding on the DNA‐Methylation Pattern in Human Adipose Tissue: A Randomized Controlled Trial1–3,” American Journal of Clinical Nutrition 105 (2017): 991–1000, 10.3945/ajcn.116.143164. 28275132

[37] S. C. Jacobsen , C. Brøns , J. Bork‐Jensen , et al., “Effects of Short‐Term High‐Fat Overfeeding on Genome‐Wide DNA Methylation in the Skeletal Muscle of Healthy Young Men,” Diabetologia 55 (2012): 3341–3349, 10.1007/s00125-012-2717-8. 22961225

[38] L. Gillberg , A. Perfilyev , C. Brøns , et al., “Adipose Tissue Transcriptomics and Epigenomics in Low Birthweight Men and Controls: Role of High‐fat Overfeeding,” Diabetologia 59, no. 4 (2016): 799–812, 10.1007/s00125-015-3852-9. 26750116

[39] S. Khatiwada , V. Lecomte , M. F. Fenech , M. J. Morris , and C. A. Maloney , “Effects of Micronutrient Supplementation on Glucose and Hepatic Lipid Metabolism in a Rat Model of Diet Induced Obesity,” Cells 10 (2021): 1751, 10.3390/cells10071751. 34359921 PMC8304500

[40] Z. Yang , R. Kubant , C. E. Cho , et al., “Micronutrients in High‐Fat Diet Modify Insulin Resistance and Its Regulatory Genes in Adult Male Mice,” Molecular Nutrition & Food Research 67 (2023): 2300199, 10.1002/MNFR.202300199. 37526337

[41] S. Khatiwada , V. Lecomte , M. F. Fenech , M. J. Morris , and C. A. Maloney , “Effects of Micronutrient Supplementation on Glucose and Hepatic Lipid Metabolism in a Rat Model of Diet Induced Obesity,” Cells 10 (2021): 8285–8296, 10.3390/CELLS10071751. PMC8304500 34359921

[42] A. A. Ribeiro , L. M. Carvalho , J. C. N. L. Da Mota , et al., “Diet, DNA Methylation, and Systemic Lupus Erythematosus: Evidence and Perspectives Focused on Personalized Nutrition,” Lifestyle Genom 17 (2024): 31–40.

[43] J. C. N. L. Da Mota , A. A. Ribeiro , L. M. Carvalho , et al., “Impact of Methyl‐Donor Micronutrient Supplementation on DNA Methylation Patterns: A Systematic Review and Meta‐Analysis of in vitro, Animal, and Human Studies,”Lifestyle Genomics 16 (2023): 192–213. 37935134 10.1159/000533193

[44] S. C. Tyagi , “A High‐Fat Diet Induces Epigenetic 1‐Carbon Metabolism, Homocystinuria, and Renal‐Dependent HFpEF,” Nutrients 17 (2025): 216, 10.3390/nu17020216. 39861346 PMC11767380

