# Supplementary material for: Genome‐Wide DNA Methylation Changes Induced by High‐Fat Diet and Methyl Donor Supplementation in Female Lupus Models: An Exploratory Study
Source: Mol Nutr Food Res. 2026 Jun 11;70(11):e70512. doi: 10.1002/mnfr.70512 (PMC13261103; doi:10.1002/mnfr.70512)
Supplement: Supplementary file 1 — Supporting File: mnfr70512‐sup‐0001‐TableS1.docx. [file MNFR-70-e70512-s001.docx]

Supplementary Table 1. Nutritional composition of the experimental diets used in the study.

| Ingredients (by kg) | Standard diet | High fat diet |
| --- | --- | --- |
| Energy (kcal) | 4,200 (4.2 kcal/g) | 6,600 (6.6 kcal/g) |
| Microcelulose | 50 | 50 |
| Sacarose​ | 100 | 100 |
| Caseína​ | 200 | 200 |
| Amido de milho​ | 397 | 197 |
| Dextrina de milho | 130.5 | 130.5 |
| Gordura vegetal hidrogenada | 0 | 200 |
| Óleo de Soja​ | 70 | 70 |
| Mix de vitaminas​ | 10 | 10 |
| Mix de minerais​ | 35 | 35 |
| L-cisteína | 3 | 3 |
| Colina | 2,5 | 2,5 |
